# DNA droplets for intelligent and dynamical artificial cells: from the viewpoint of computation and non-equilibrium systems

**DOI:** 10.1098/rsfs.2023.0021

**Published:** 2023-08-11

**Authors:** Masahiro Takinoue

**Affiliations:** ^1^ Department of Computer Science, Tokyo Institute of Technology, 4259 Nagatsuta-cho, Midori-ku, Yokohama, Kanagawa 226-8502, Japan; ^2^ Department of Life Science and Technology, Tokyo Institute of Technology, 4259 Nagatsuta-cho, Midori-ku, Yokohama, Kanagawa 226-8502, Japan; ^3^ Living Systems Materialogy (LiSM) Research Group, International Research Frontiers Initiative (IRFI), Tokyo Institute of Technology, 4259, Nagatsuta-cho, Midori-ku, Yokohama 226-8501, Japan

**Keywords:** DNA droplet, DNA coacervate, artificial cell, molecular computer, non-equilibrium open system, protocell

## Abstract

Living systems are molecular assemblies whose dynamics are maintained by non-equilibrium chemical reactions. To date, artificial cells have been studied from such physical and chemical viewpoints. This review briefly gives a perspective on using DNA droplets in constructing artificial cells. A DNA droplet is a coacervate composed of DNA nanostructures, a novel category of synthetic DNA self-assembled systems. The DNA droplets have programmability in physical properties based on DNA base sequence design. The aspect of DNA as an information molecule allows physical and chemical control of nanostructure formation, molecular assembly and molecular reactions through the design of DNA base pairing. As a result, the construction of artificial cells equipped with non-equilibrium behaviours such as dynamical motions, phase separations, molecular sensing and computation using chemical energy is becoming possible. This review mainly focuses on such dynamical DNA droplets for artificial cell research in terms of computation and non-equilibrium chemical reactions.

## 1. Introduction

**Figure 1.**
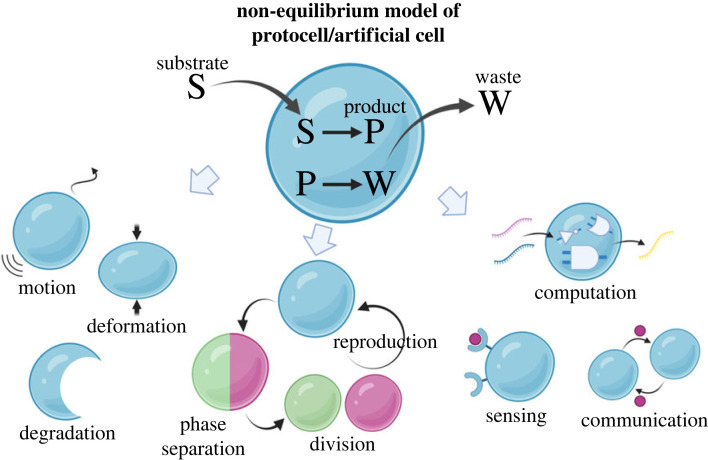
Concept of non-equilibrium artificial cells showing various dynamics and functions such as autonomous motion, deformation, division, self-reproduction and computation.

The basic building blocks of living systems are cells. Cells are micrometre-sized molecular assemblies whose dynamics are maintained by non-equilibrium chemical reactions and physical phenomena of biological soft matter. For example, an immune cell, macrophage, can sense bacteria, virus-infected cells and tumour cells, and phagocytose such enemies using non-equilibrium molecular reaction networks and fluidic motion and deformation of biological soft matter in its cell [1,2]. Another example is a cell nucleus: a cell appropriately separates replicated chromosomes and divides the nucleus synchronously with the cell division [1,2]. The generation of such sophisticated dynamical behaviours generally requires non-equilibrium chemical reaction systems [3] with a soft, deformable reaction field, where energy, matter and information flows into and out of the soft reaction field are sustained. So far, many studies have been reported on non-equilibrium protocell models (figure 1) [4–7] and molecular robots inspired by cell-like dynamical behaviours [8,9]. There are two types of systems for artificial cells: (i) ‘membranous' molecular assemblies using lipid bilayer vesicles (liposomes) or water-in-oil droplets [4–7,10–13] and (ii) ‘membrane-less' molecular assemblies using hydrogels, biomolecular coacervates (a form of associative liquid–liquid phase-separated (LLPS) droplets), or segregative LLPS droplets [14–19]. In both cases, the goal is to create a dynamical artificial cell with autonomous functions based on non-equilibrium chemical reaction systems.

## 2. Molecular computing and informatics aspects of artificial cells

**Figure 2.**
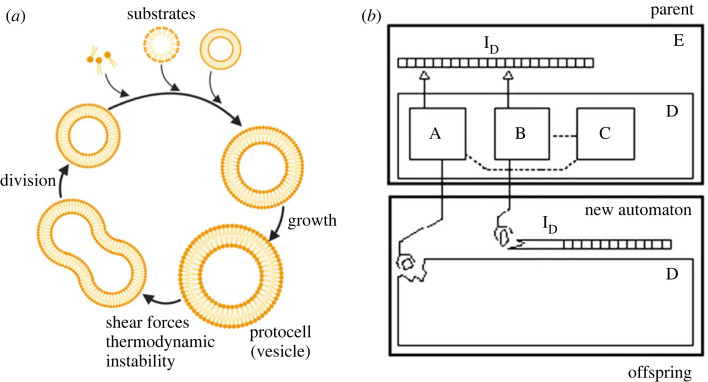
Protocell models. (*a*) Protocell model of a self-replicating vesicle under a non-equilibrium condition proposed by Szostak *et al*. [4]. (*b*) Von Neumann's self-reproducing automaton (self-replicating machine). A: universal constructor. B: Copier. C: Controller. I_D_: Instruction tape. D: Hardware including A, B and C. E: Software. Image adapted from [6]. Copyright 2011 National Academy of Sciences.

Although replicating the body under a non-equilibrium condition (figure 2*a*) [4] is important for life, replicating information is also essential. The self-reproducing automaton (self-replicating machine) proposed by John von Neumann (figure 2*b*) [22,23] gives a perspective of life from an informatics viewpoint [5,6]. The self-reproducing automaton comprises hardware (D in figure 2*b*) and software (E); the hardware consists of a universal constructor (A), copier (B) and controller (C). The software has an instruction tape (I_D_), which plays the roles of code and program. The universal constructor builds the offspring's hardware by following the command of the instruction tape. The copier duplicates the instruction tape. The controller controls the universal constructor and the copier. The offspring replicates itself again in the same way. In biological systems, the instruction tape is genetic information such as DNA; non-equilibrium chemical reaction networks realize the universal constructor, copier and controller. Thus, the molecular program coded with information molecules and the non-equilibrium chemical reaction networks are the essence of an autonomous and dynamical artificial cell or protocell model; such systems must be encapsulated in a body, such as a lipid vesicle or a coacervate.

Regarding von Neumann's self-reproducing automaton, DNA would be one of the promising materials for building artificial cells and protocell models. DNA can work as both hardware and software: DNA can realize non-equilibrium chemical reaction networks, even like replication based on molecular programs on DNA. Although, at this moment, the functions of the universal constructor and the copier require catalytic activities provided by enzymes or organic/inorganic catalysts in addition to DNA molecules, the programmability of DNA would be essential to design and create artificial cells in a programmable manner.

DNA has high molecular recognition ability, achieved by specific Watson–Crick base pairing. The thermodynamic stability of base pairing can be estimated based on free energy calculations using the nearest-neighbour model [24], which allows the prediction of intramolecular secondary structure formation and intermolecular association reactions from base sequences with high accuracy; e.g. the Nucleic Acid Package (NUPACK) is available for this purpose [25]. Therefore, self-assembled DNA nanostructures can be precisely designed and controlled at the nanometre level based on sequence information, and this concept has been extended to even micro- to millimetre scales [26–29]. In DNA nanotechnology, this property is called the programmability of DNA. In addition, DNA nanostructures can be chemically modified by functional materials such as RNA aptamers, enzymes and metal nanoparticles. With these advantages, DNA nanostructures are often used for various applications, such as drug delivery systems and nanodevices for cancer diagnosis in cells [30,31].

**Figure 3.**
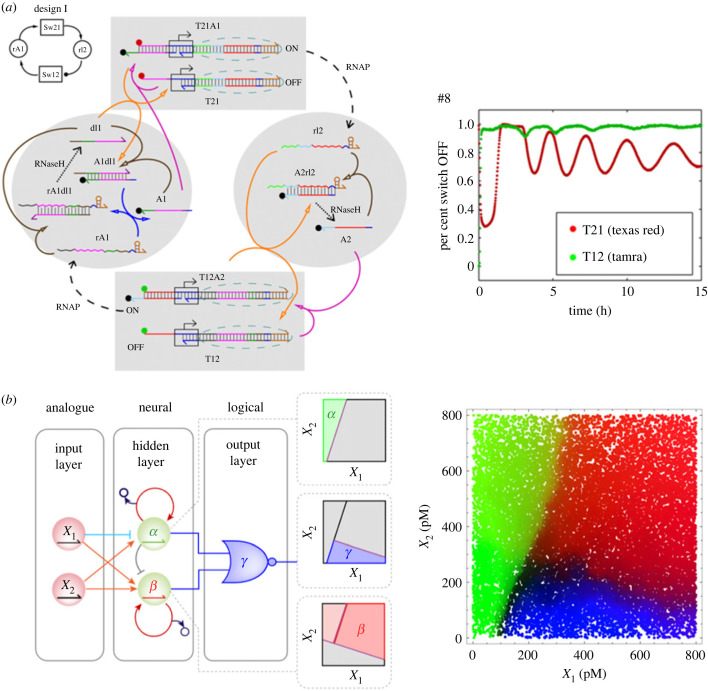
Dynamical systems by molecular computing systems. (*a*) Biochemical oscillator. Images adapted from [39] licensed under Creative Commons (CC BY-NC-ND). (*b*) Enzymatic neural networks for nonlinear decision-making. Images adapted from [51] with permission. Copyright 2022 Springer Nature.

Furthermore, Leonard Adleman demonstrated that DNA molecules could perform mathematical calculations [32]. This technique is called molecular computing (DNA computing). Initially, Adleman considered DNA molecules as computational elements; he generated solution candidates for the directed Hamiltonian path problem by spontaneous DNA hybridization and extracted a proper solution using molecular biology techniques. Subsequently, Shapiro's group realized a molecular automaton (autonomous DNA computer) that accepts messenger RNAs (mRNAs) as input and releases DNA outputs that function as a drug [33,34]. After that, the DNA computing field has been vigorously pioneering autonomous DNA computers that can perform logical operations based on designed molecular reaction circuits, just as biological systems realize genetic circuits and neural networks based on complex molecular reaction networks of multiple DNA/RNA strands and enzymes [35–53]. Winfree's group has demonstrated enzyme-free autonomous DNA computation systems using nucleic acid hybridization and strand displacement reactions [35–37], as well as enzyme-assisted autonomous DNA computation systems [38,39] (figure 3*a*). The enzyme-free DNA molecular computing circuits were extended to more complex dynamical systems [40,41]. Suyama's group proposed a reverse transcription and transcription-based autonomous computing system accepting RNA inputs and generating RNA outputs, modelled on retroviral replication [42,43,55]. Rondelez's group proposed the PEN-DNA toolbox system [44–46] as a universal method to create dynamical systems like biological systems based on enzyme-assisted DNA reaction circuits. Reif's group recently showed fast, compact logic circuits using strand-displacing polymerase [47]. These results showed that designed artificial complex molecular reaction networks using DNA could mimic the dynamics of biological systems. Recent progress in the DNA computing field has reached the development of artificial cells with dynamical reaction circuits showing oscillation [48,49] and artificial neural networks (figure 3*b*) [50,51]. Thus, DNA computing has a significant advantage in artificial cell construction in designing molecular inputs and outputs and controlling other molecular systems based on nonlinear non-equilibrium chemical reactions.

## 3. DNA droplets

Artificial micrometre-sized self-assemblies of DNA nanostructures are well-investigated these days. DNA hydrogels made of branched DNA nanostructures can self-assemble and exhibit various functions, such as molecule encapsulation ability, RNA transcription ability and mechanical/viscoelastic properties [28,56–58]. There are two types of DNA hydrogels: (i) chemical gels [28], in which DNA nanostructure monomers are covalently bonded by ligation and (ii) physical gels, in which association and dissociation are reversible due to weak interactions such as base pairing and entanglement [57,59].

**Figure 4.**
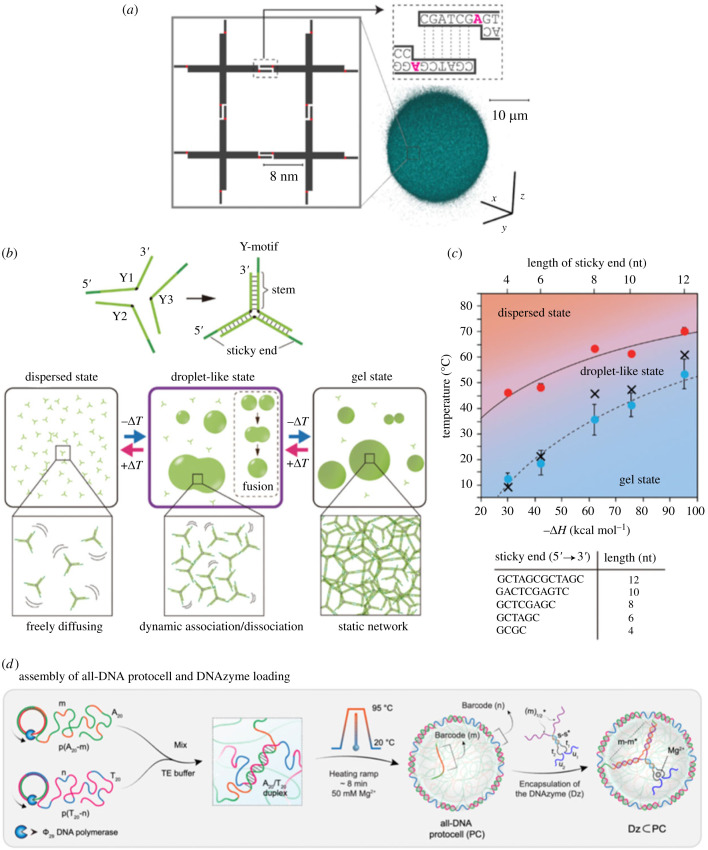
DNA droplets. (*a*) DNA droplets formed from branched DNA nanostructures. Images adapted from [20] with permission. Copyright 2019 American Chemical Society. (*b*) State transition of DNA droplets based on branched DNA nanostructures. (*c*) Dependence of the state transition on the sticky-end length. (*b*,*c*) Images adapted from [21]. (*d*) DNA protocells formed from long ssDNA produced by a RCA. Images adapted from [54] licensed under Creative Commons (CC BY).

In cell biology, biomolecular assemblies by reversible and weak bonding, such as hydrogen bonding and π–cation interaction, are known as intracellular LLPS droplets [60] and have recently attracted much biological attention as they are thought to be involved in various cellular dynamics, such as expression and regulation of biochemical reactions [60–63]. Because of the reversible and weak bonds, LLPS droplets have dynamical properties that change their state and function in response to the environment, including temperature, pH, ionic strength and biomolecules. Similarly, DNA-based physical gels also have the property of shifting their structure from a gel state to a liquid-like state by responding to the environment thanks to the reversible binding (figure 4*a*) [20,21,64,66–69].

Recently, several research groups have constructed ‘DNA droplets’. The DNA droplets are lipid-membrane-less coacervates, which have been vigorously applied to membrane-less protocells and membrane-less organelles controlled by DNA sequence information. Mainly, two types of DNA droplets have been reported. The first one is DNA-based coacervates (figure 4*a–c*) generated by the self-assembly of branched DNA nanostructures (also referred to as DNA nanostars or branched DNA motifs) such as Y- and X-shaped nanostructures (figure 4*b*, top). When the appropriately designed branched DNA nanostructures are cooled down from a high temperature (approx. 95°C), the branched DNA nanostructures make a network to form fluid DNA droplets at around 60°C (figure 4*b*, bottom middle); finally, the fluid DNA droplets transition to a non-fluid DNA gel around room temperature (figure 4*b*, bottom right) (however, the transition temperatures are affected by experimental conditions). The dispersion–droplet and droplet–gel transition temperatures depend on the DNA sequences (figure 4*c*) [21] and ionic strength [69]. In addition, the interfacial tension of DNA droplets was 0.01–4 µN m^−1^ [69,70], which is significantly lower than the air–water interfacial tension, and their viscosity was several tens of Pa s [69]; these physical properties also could be changed by the sticky-end length [70]. Historically, branched DNA nanostructures were proposed by Seeman to create ordered phases like crystals [26,71,72], but later, the flexible, simple star-like motifs were found not to form an ordered phase because gel/coacervate-like disordered phases are thermodynamically the most stable [73]; i.e. the flexible star-like motifs are found to be suitable for the formation of DNA gels/droplets, although more rigid branched structures are required to form DNA crystals [74].

The physical properties of DNA droplets based on branched nanostructures, such as their stability and specificity, are controlled not only by the sticky-end sequences of the branched nanostructures but also by nanomechanical and physico-chemical properties of DNA nanostructures, such as the number of branches; the shape, size and flexibility of the nanostructures; and amphiphilicity of the chemically modified DNA nanostructures [21,64,65,67,70,75–78]. For example, if the number of branches changed from 3 to 6 arms, the gel–droplet transition temperature rose about 30°C in the case of the 8-nucleotide sticky end [21]. When the arm length of the Y-shaped DNA nanostars increased from 8 to 40 base pairs, the average size of the resultant DNA droplets also increased [77], which shows that the growth kinetics of DNA droplets was affected by the arm length.

The other DNA droplet is DNA-based coacervates constructed by hybridizing and entangling polymerized long single-stranded DNAs (ssDNAs) produced using rolling circle amplification (RCA) (figure 4*d*) [54,59,79,80]. The formation mechanism differs between RCA-based DNA droplets and those based on branched DNA nanostructures. After the synthesis of long ssDNAs with the RCA reaction, the temperature increase induced the demixing and entanglement of the long ssDNAs; this physico-chemical property of polymers is called a lower critical solution temperature-type demixing. Then, by decreasing temperature, the entangled state is fixed by the hybridization between the long ssDNAs. The physical and chemical properties of RCA-based DNA droplets are also tunable by sequence designing. Using this method, Walther's group has realized DNA-based protocells.

## 4. Non-equilibrium dynamics of DNA droplets

### 4.1. Controlled fusion, division and pattern formation dynamics of DNA droplets

**Figure 5.**
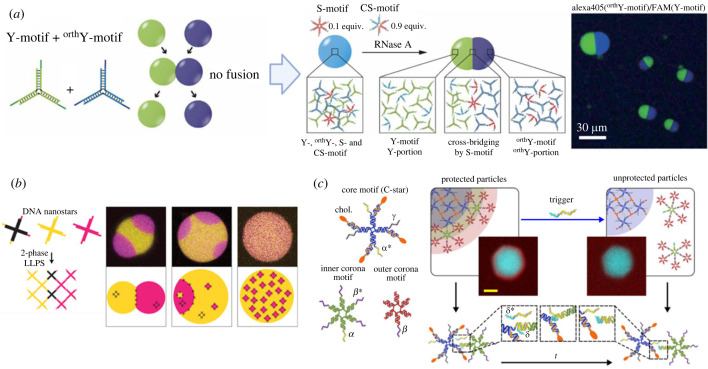
Multi-compartment structures of DNA droplets. (*a*) Janus structure. Images adapted from [21]. (*b*) Multi-compartment and emulsion-like structures. Images adapted from [64] with permission. Copyright 2020 American Chemical Society. (*c*) Core–shell structure. Images adapted from [65] licensed under Creative Commons (CC BY).

The dynamical behaviours of DNA droplets, such as fusion, division and pattern formation, can be controlled by the sequence of the sticky ends and the nanomechanical properties of branched DNA nanostructures [21,64]. For example, DNA droplets fused when composed of DNA nanostructures with complementary sticky ends; they did not fuse in other cases (figure 5*a*) [21]. Even if the branched DNA nanostructures did not directly make fusion, they could make Janus DNA droplets (figure 5*a*) [21] and multi-compartment droplets (figure 5*b*) [64] with linker DNAs. In addition, Di Michele's group has successfully generated core–shell structures of DNA droplets/gels [65] in which an amphiphilic branched DNA nanostructure core was coated with other branched DNA nanostructures (figure 5*c*); they also demonstrated the triggered release of the DNA shell (figure 5*c*).

In these examples, the linker DNAs act as programmable, dynamical ‘surfactants' that can control the ‘amphiphilicity’ between two or more types of DNA nanostructures. The design of the linker amphiphilicity is currently based on the sticky-end hybridization stability, the multi-valency of the linker branches, and the branch structures and flexibility; however, the design is still in an ad hoc manner. If more sophisticated design principle is established and some kinds of design software are developed, this field will proceed faster. Another interesting feature of DNA droplets is that relatively weak interaction of the sticky ends (only several nucleotides) decides the behaviours of DNA droplets. This phenomenon would be related to the behaviour of the above-mentioned LLPS droplets of biomolecules in actual living cells. The remarkable feature of artificial DNA droplets compared to the natural LLPS droplets is that the DNA droplets are well controlled based on reversible, weak interactions embedded as base sequence information. The design of reversible, weak binding would be a different concept of DNA sequence design from DNA nanostructure research and DNA computing driven by the design of stable hybridization. The advantage of DNA droplet systems is that DNA sequences can control micrometre-sized (i.e. cell-sized) self-assembly processes, as shown; this feature will strongly promote the construction of dynamical artificial cells.

### 4.2. Non-equilibrium dynamics of DNA droplets using enzymatic reactions

**Figure 6.**
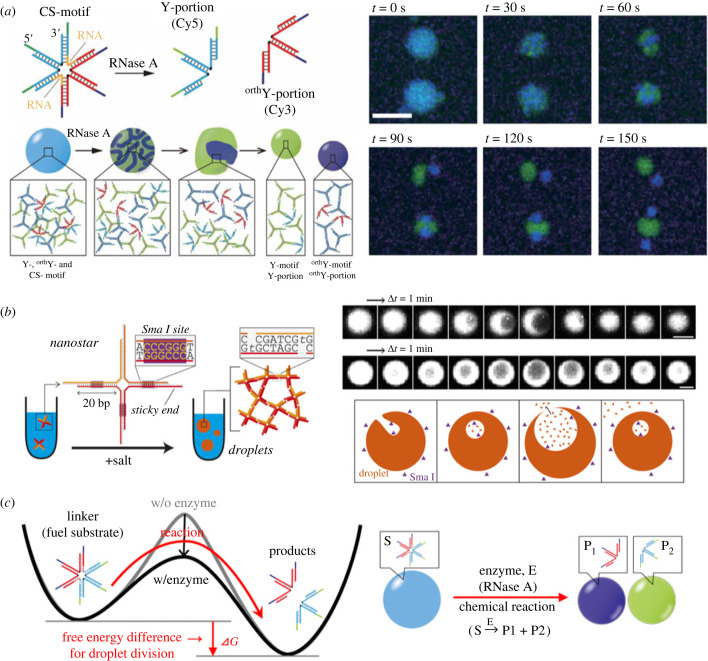
Dynamical DNA droplets with enzymatic reactions. (*a*) Division of DNA droplets. Images adapted from [21]. (*b*) Bubbling of a DNA droplet by the degradation with a restriction enzyme. Image adapted from [81]. Copyright 2021 National Academy of Sciences. (*c*) Thermodynamic description of the division of DNA droplets with enzymatic cleavage of the linker.

DNA droplets can show dynamics by recognizing environmental biomolecular information such as proteins and RNAs or by coupling with non-equilibrium biomolecular reactions. We fabricated a six-branched DNA nanostructure that can bridge two different Y-shaped DNA nanostructures [21]; three arms of the six-branched DNA nanostructure bind one of the Y-shaped DNA nanostructures and another three arms bind the other Y-shaped DNA nanostructures (figures 5*a* and 6*a*), allowing the blue and green DNA droplets to fuse. Here, since the six-branched DNA nanostructures were designed to have RNA parts at their branch centre, the six-branched DNA nanostructures can recognize the presence of ribonuclease (RNase) and split into two portions, which induced the division of the DNA droplet into two DNA droplets (figure 6*a*, blue- and green-coloured DNA droplets). The repulsion of DNA drove the droplet phase separation because of no association with one another. Saleh's group has also demonstrated the dynamical properties of DNA droplets by incorporating enzymatic reactions (figure 6*b*) [81]. They found the bubbling phenomenon of DNA droplets by sequence-specific cleavage of branched DNA nanostructure arms with a restriction enzyme, which showed the dissolution of DNA droplets from the inside (figure 6*b*). From a thermodynamic viewpoint, the non-equilibrium dynamics with the enzymes to show droplet division or bubbling was caused by the free energy difference in the chemical bond of the RNA or DNA backbone. Figure 6*c* shows the free energy landscape to explain the division dynamics by RNase reaction. In this reaction, the six-branched linker DNA also plays a role of a fuel substrate. The RNase cleaves the six-branched DNA and produces the free energy difference between the six-branched DNA and the cleaved DNA nanostructures; finally, DNA droplets are divided using the free energy difference.

**Figure 7.**
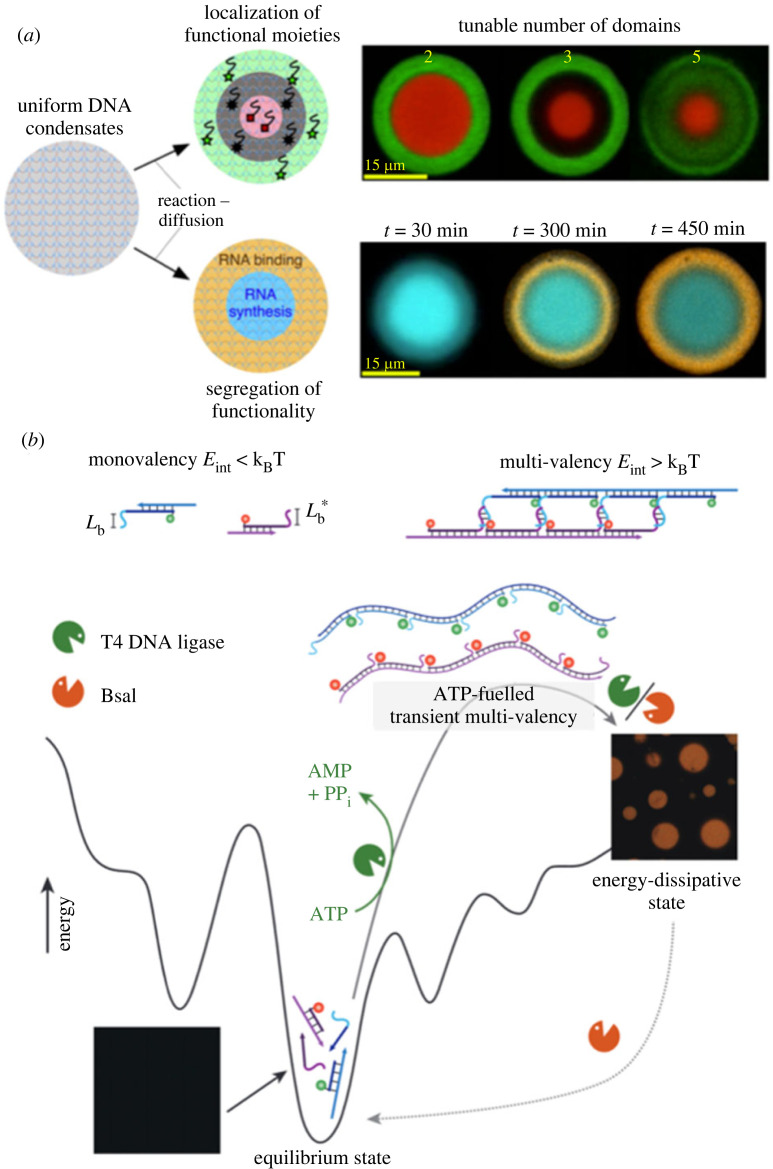
Non-equilibrium DNA droplets. (*a*) Reaction–diffusion pattern formation in DNA droplets. Images adapted from [82] licensed under Creative Commons (CC BY). (*b*) Transient LLPS of DNA coacervates with ATP fuel. Images adapted from [83] with permission. Copyright 2020 Elsevier Inc.

Di Michele's group has also demonstrated that the core–shell DNA droplets produced using the amphiphilic branched DNA nanostructures dynamically collapsed lipid membranes of liposomes and captured bacteria [65]. Additionally, they realized a reaction–diffusion pattern in DNA droplets with the core–shell structures (figure 7*a*) [82]. Walther's group generated DNA droplets as a non-equilibrium steady state by coupling the synthesis of branched DNA nanostructures by ligation using ATP energy and the degradation by restriction enzyme cleavage (figure 7*b*) [83]. Walther's non-equilibrium DNA droplets are entirely different from other equilibrium DNA droplets in the free energy minimum; thus, they will provide a new basis for constructing non-equilibrium artificial cells. In addition, Hamada *et al*. demonstrated the autonomous locomotion of RCA-based DNA assemblies by combining them with enzymatic synthesis and degradation reactions mimicking metabolic reactions [87].

In all examples, chemical energy, such as ATP and DNA/RNA chemical bonding energy, is converted to non-equilibrium dynamics. How the free energy change is converted to the actual non-equilibrium dynamics is programmed in DNA droplets. In the next step, the complexity of molecular programs will be the point of research: for example, feedback loops and multistep cascades of dynamics.

### 4.3. Molecular computation by DNA droplets

**Figure 8.**
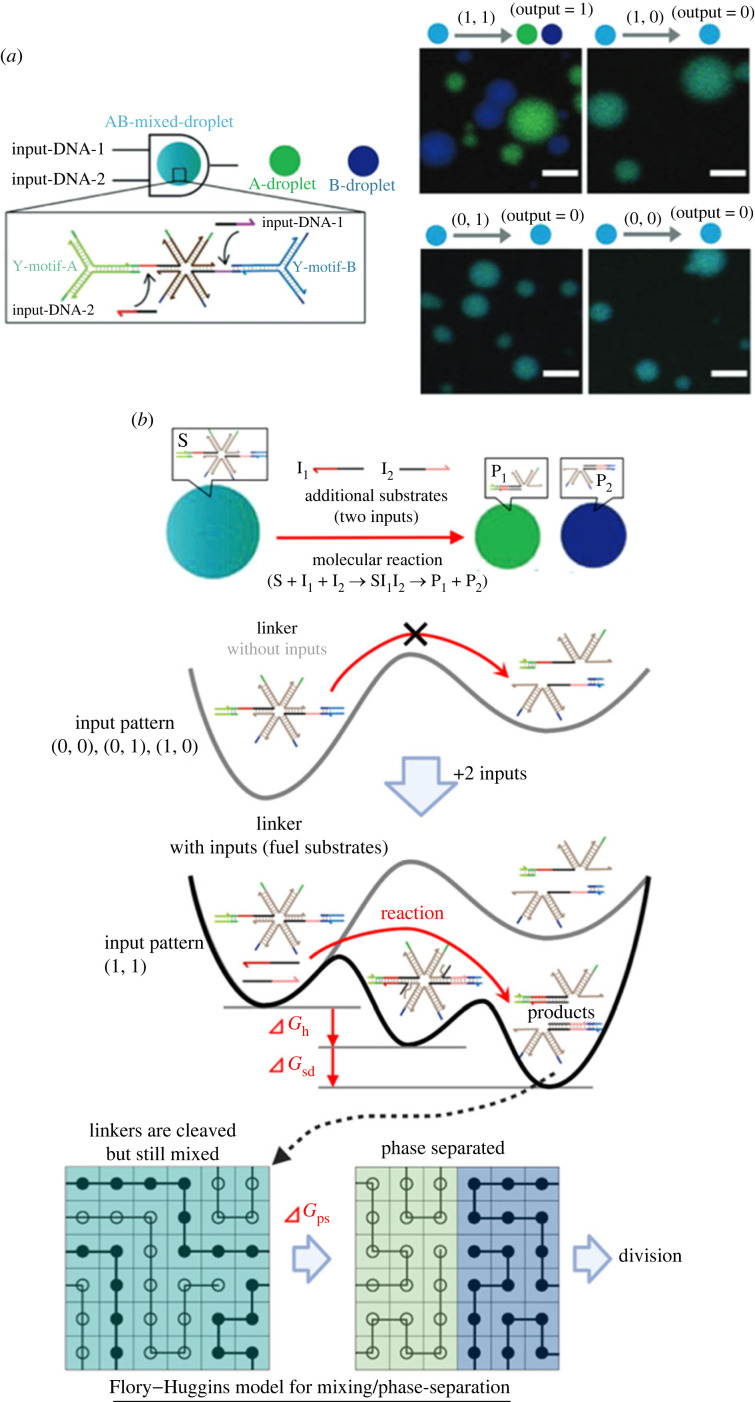
Computational DNA droplets. (*a*) AND gate. Images adapted from [84]. (*b*) Thermodynamic description of the AND gate. Two miRNA inputs are used as a substrate to perform the cleavage reaction of the linker DNA. By the hybridization and their strand displacement of two inputs, the free energy decreased by $\Delta G_{h}+\Delta G_{\mathrm{sd}}$. Just after the linker cleavage, the DNA droplet is almost mixed (bottom left); then, phase separation occurs with the free energy decrease $\Delta G_{\mathrm{ps}}$ (bottom right), resulting in DNA droplet division.

A DNA droplet-based logic computer is one example of more complex DNA droplet systems using non-equilibrium dynamics. The DNA droplet computer can be constructed by adding logical operation capabilities to branched DNA nanostructures. To recognize biomarker microRNAs (miRNAs), a six-branched DNA droplet was modified to have ssDNA parts for miRNA binding [84]. After the hybridization of two input miRNAs (*I*_1_ and *I*_2_) to the six-branched DNA nanostructures, strand displacement reactions by the two miRNAs caused cleavage; then, DNA droplets were divided, demonstrating an AND operation (*I*_1_ ∧ *I*_2_) (figure 8*a*). This system was extended to a logic calculation for four RNA inputs: (*I*_1_ ∧ *I*_2_) ∧ (*I*_3_ ∧ ¬*I*_4_).

The logical computation was driven by the free energy difference $\Delta G_{\mathrm{total}}=\Delta G_{h}+\Delta G_{\mathrm{sd}}+\Delta G_{\mathrm{ps}}$, where $\Delta G_{h}$, $\Delta G_{\mathrm{sd}}$ and $\Delta G_{\mathrm{ps}}$ are the free energy differences due to the DNA hybridizations, the strand displacement reactions and the mixing/phase separation of two types of branched DNA nanostructures, respectively (figure 8*b*). $\Delta G_{h}$ and $\Delta G_{\mathrm{sd}}$ can be estimated by the nearest-neighbour model with SantaLucia's parameters [24]; $\Delta G_{\mathrm{ps}}$ can be estimated based on the Flory–Huggins theory for polymer phase separation [60]. Figure 8*b* shows a thermodynamic view of this dynamical reaction system. Adding miRNAs changes the free energy landscape, and non-equilibrium molecular reactions for computation occur autonomously. The unique aspect of DNA droplet computing is that it also exploits the free energy difference between the mixing and phase separation of DNAs as polymers. Like this, in DNA droplet computing, the soft matter physics of DNA would play an essential role in the design of the system as well as the physical chemistry of DNA.

Currently, the output of this system has been limited to displaying the computation results as the droplet division; in the next step, it should be improved to emit the outputs of computation results as information molecules such as DNA, RNA and proteins to achieve the logic circuits of the DNA droplet logic gates. If logic circuits with input–output cascades are realized, this system will get more valuable in creating intelligent artificial cells. In the future, introducing more complex logic gates and integrating multiple inputs and outputs for molecular information processing may lead to applications in the early detection of diseases and regenerative therapies.

### 4.4. Non-equilibrium dynamics of DNA droplets in a cell-mimicking tiny space

**Figure 9.**
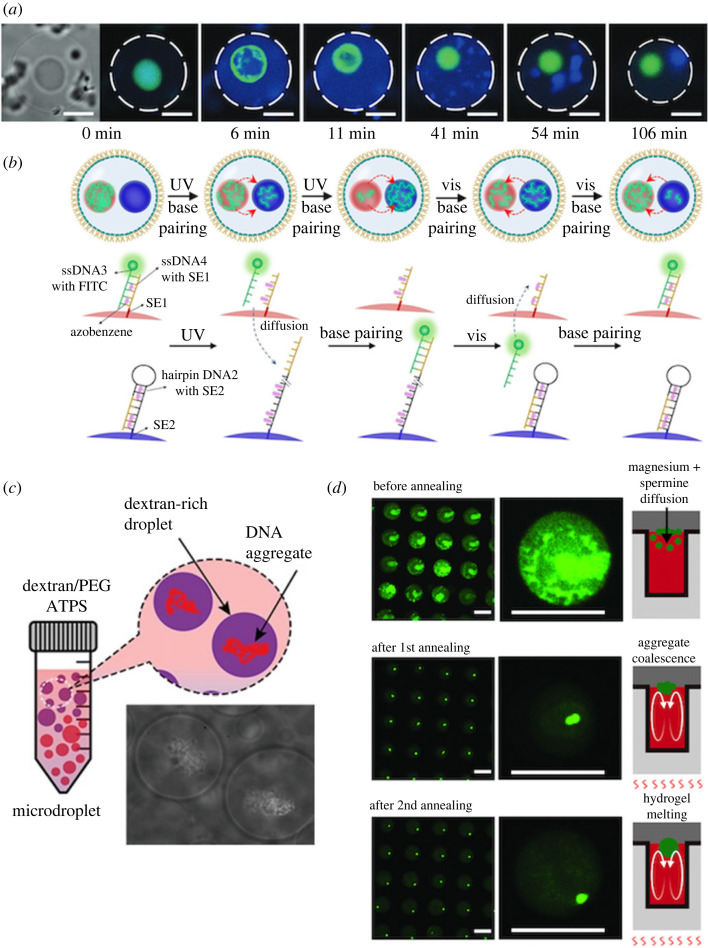
DNA droplets in a cell-sized tiny space. (*a*) DNA droplet division in a giant unilamellar lipid vesicle. Images adapted from [85] licensed under Creative Commons (CC BY). (*b*) Molecular communication between DNA droplet-based artificial organelles. Images adapted from [86] with permission. Copyright 2022 Wiley-VCH GmbH. (*c*) Dextran-in-PEG droplets encapsulating aggregates of salmon sperm DNAs. Images adapted from [88] licensed under Creative Commons (CC BY). (*d*) Formation of monodisperse DNA droplets in dextran-in-PEG droplets by convection under a thermal gradient. Scale bars: 100 µm. Images adapted from [89].

Here, we describe the control of DNA droplets through the interactions with a cell-sized tiny space or those with environmental polymers as challenges of applying DNA droplets to artificial cells and organelles. Göpfrich's group has constructed an artificial cell model encapsulating DNA droplets in a water-in-oil (W/O) microdroplet [85] (figure 9*a*). In this case, the spatially confined DNA droplets were appropriately divided into two droplets because some divided DNA droplets were re-fused with one another in the tiny space of W/O microdroplets. DNA coacervates constrained at the interface of oil-in-water droplet emulsions and liposomes exhibit phase-separation patterns [90]. Deng's group also proposed DNA droplets as artificial membrane-less organelles and has demonstrated molecular communication between the artificial organelles (figure 9*b*) [86]. In the future, the spatial organization of artificial organelles based on DNA droplets confined in a tiny space will be explored, similar to polymer gel-based artificial organelles [91].

In addition, the intracellular environment is not a dilute aqueous solution but an environment filled with highly condensed water-soluble polymers. Aqueous two-phase systems of dextran and polyethylene glycol (PEG) are often used to mimic such an intracellular environment. In particular concentration conditions, dextran microdroplets form in a continuous phase of PEG solution by microphase separation. Yoshikawa's group found that genome-sized DNAs were incorporated into dextran droplets in a size-dependent manner and could be used as artificial cells (figure 9*c*) [88]. We showed that DNA droplets were also incorporated into the dextran microdroplets [89], where uniform-sized DNA droplets were generated in cell-sized microchambers (approx. 100 µm) in a microchannel. A temperature gradient was created between the top and bottom of the microchamber, causing non-equilibrium convection in the microchamber, and the convection flow collected the DNA droplets into one (figure 9*d*). The growth of phase-separated biomolecular droplets in the presence of a thermal gradient and convection may relate to the research on the origins of life.

## 5. Discussion and conclusion

In this review, we introduced a new technology, DNA droplet (DNA coacervate), and gave a perspective on its application to constructing artificial cells with intelligence and non-equilibrium dynamics. The DNA droplet is a new programmable biomacromolecular coacervate that can exhibit intelligent and dynamical behaviours by converting chemical energy to sensing [84], computation [84] and motion [87] functions. However, examples are still limited, although vigorous related research is ongoing [21,54,81–87]. The current challenges and future directions will be summarized as follows.

From the viewpoint of basic science in physical chemistry and information nanotechnology, the dynamical systems of DNA droplets using chemical energy should be explored more in the next step. For example, constructing more complex logic circuits or reaction networks (cascade reactions) including a nonlinear response function would be highly desired. Non-equilibrium dynamical motions of DNA droplets also would be desired because there are only a few examples, such as locomotion [87] and division [21]. In addition, remote control of DNA droplets with magnetic/electrical fields or light irradiation would be valuable in this field. The encapsulation of DNA droplets to mimic organelles in an artificial cell would help to understand the effect of the cell-sized space for biochemical reactions. From the viewpoint of applications, the combination between DNA droplets and other targets would be essential. For example, the interaction of DNA droplets and living biological cells has not been reported except for an example by Di Michele's group [65]. The recognition of cell surfaces or the introduction of DNA droplets into a cell to control transcription and translation of the cell will be a challenging topic. These directions are essential for DNA droplet research to diagnosis and therapy.

Various LLPS droplets have recently been found in living cells and have attracted attention because of their essential roles in living systems in the control of inter-/intramolecular reactions and soft matter physical phenomena [60–63]. Combining such biophysical phenomena in cells with programmable DNA droplets would be a practical approach to constructing intelligent molecular systems and a novel cell control technology. In the future, coupling the self-dividing DNA droplets and gene expression functions or amplification will lead to the construction of artificial cells and artificial organelles autonomously working under a non-equilibrium environment. The artificial cells and artificial organelles constructed with programmable DNA droplets differ from existing living systems in terms of molecular compositions; however, they give us universal design principles and minimum elements of living systems in terms of physics, chemistry and informatics. In particular, the programmability of DNA would help us construct artificial cells from an informatics perspective, such as the idea of a self-reproducing automaton [5,6,22,23]. At the same time, they would be valuable in various fields: they will lead to the construction of molecular robots, molecular computers and other applications such as cancer diagnosis, treatment in living cells and novel molecular-electrical devices.

## Data Availability

This article has no additional data.
